# Identification of Novel Clinical Factors Associated with Hepatic Fat Accumulation in Extreme Obesity

**DOI:** 10.1155/2014/368210

**Published:** 2014-12-24

**Authors:** Glenn S. Gerhard, Peter Benotti, G. Craig Wood, Xin Chu, George Argyropoulos, Anthony Petrick, William E. Strodel, Jon D. Gabrielsen, Anna Ibele, Christopher D. Still, Christopher Kingsley, Johanna DiStefano

**Affiliations:** ^1^Department of Medical Genetics and Molecular Biochemistry, Temple University School of Medicine, 3500 N. Broad Street, Philadelphia, PA 19140, USA; ^2^Geisinger Obesity Research Institute, Danville, PA 17822, USA; ^3^Translational Genomics Institute, Phoenix, AZ 85004, USA

## Abstract

*Objectives*. The accumulation of lipids stored as excess triglycerides in the liver (steatosis) is highly prevalent in obesity and has been associated with several clinical characteristics, but most studies have been based on relatively small sample sizes using a limited set of variables. We sought to identify clinical factors associated with liver fat accumulation in a large cohort of patients with extreme obesity. *Methods*. We analyzed 2929 patients undergoing intraoperative liver biopsy during a primary bariatric surgery. Univariate and multivariate regression modeling was used to identify associations with over 200 clinical variables with the presence of any fat in the liver and with moderate to severe versus mild fat accumulation. *Results*. A total of 19 data elements were associated with the presence of liver fat and 11 with severity of liver fat including ALT and AST, plasma lipid, glucose, and iron metabolism variables, several medications and laboratory measures, and sleep apnea. The accuracy of a multiple logistic regression model for presence of liver fat was 81% and for severity of liver fat accumulation was 77%. *Conclusions*. A limited set of clinical factors can be used to model hepatic fat accumulation with moderate accuracy and may provide potential mechanistic insights in the setting of extreme obesity.

## 1. Introduction

Obesity is associated with fat accumulation in the liver, diagnosed as nonalcoholic fatty liver disease, and considered a manifestation of the metabolic syndrome [1, 2]. Although the relationship between obesity and fatty liver is highly interconnected, the pathogenic mechanisms linking obesity and the accumulation of lipids in the liver are poorly understood. For example, hepatic lipid accumulation has been proposed as a cause, rather than a consequence, for the development of hepatic insulin resistance [3], but the molecular mechanism by which this occurs is not yet known.

Both environmental and genetic factors play a significant role in mediating risk for development of increased fat in the liver [4]. A major environmental determinant is obesity, and extreme obesity in particular is associated with high prevalence of hepatic fat accumulation. For example, in a meta-analysis of patients with severe obesity undergoing bariatric surgery, the prevalence of histological evidence of hepatic lipid accumulation was 85–98% [5]. Fatty liver has generally been considered to be a benign condition, although it may influence the expression of enzymes involved in drug metabolism [6], and has been hypothesized to play a primary role in the pathogenesis of type 2 diabetes [7]. Liver fat accumulation is also clinically important because a substantial subset of patients will develop inflammation as nonalcoholic steatohepatitis (NASH) with necroinflammatory changes that can progress to fibrosis, cirrhosis, and liver failure [8]. NASH-related cirrhosis has a 10-year survival rate comparable to hepatitis due to HCV infection [9] and is responsible for approximately one-third of all liver transplant operations in the USA [10]. Defining the clinical context in which hepatic fat accumulates in extreme obesity may thus have significant implications for several clinically important conditions.

The clinical standard for diagnosis and staging of hepatic fat is liver biopsy [11]. Such data from deceased transplant donors suggest that about half have some degree of hepatic lipid [12], and one of the most common reasons for disqualification of living hepatic transplant donors is excess fat [13]. Few large studies have been conducted with histologically documented liver fat and most studies have also been restricted to a relatively limited number of common clinical variables, thus limiting statistical power resulting in few robustly associated variables [14]. Here, we analyzed a large cohort of patients with extreme obesity undergoing intraoperative liver biopsy during a primary bariatric surgery to identify clinical factors associated with fatty liver. We conducted an association analysis of over 200 clinical variables with presence or severity of hepatic fat accumulation and then used multiple regression to identify a set of independently associated variables. This analysis represents the largest and most complete clinicopathological study of fatty liver yet performed.

## 2. Subjects and Methods

### 2.1. Subjects

Patients enrolled in the Bariatric Surgery Program at the Geisinger Clinic Center for Nutrition and Weight Management were offered participation in an IRB approved research program on obesity. Clinical data were obtained during a standardized in-depth preoperative preparation program as described [15]. Participants who had a primary bariatric surgery performed between 9/1/2002 and 2/28/2013, preoperative BMI >35 kg/m^2^, no evidence of hepatitis B virus (HBV), hepatitis C virus (HCV), or human immunodeficiency virus (HIV), evaluable liver biopsy results, and no diagnosis of alcohol abuse were included in this study. Alcohol abuse was evaluated during a required comprehensive behavioral evaluation with a clinical psychologist prior to surgery, where patients were asked about current and past substance and alcohol use. If a patient endorsed using alcohol, they were further queried based on the clinical criteria for alcohol use disorders [16]. Patients who met the clinical criteria for alcohol use disorders were denied access to bariatric surgery. All patients with EHR diagnosis codes indicating a clinical diagnosis of alcohol abuse, ICD9 303 or ICD9 305.0, were also excluded. For data collection and analysis, alcohol use (not abuse) was defined as any alcohol intake, regardless of amount, that was recorded during the preoperative clinical and psychological evaluation.

Wedge biopsies of the liver, obtained intraoperatively during the bariatric surgery from a similar anatomic location as part of clinical standard of care [17], were fixed in neutral buffered formalin and stained with hematoxylin and eosin for histological evaluation of steatosis using NASH CRN criteria [18]. A second pathologist reviewed histological data. For histological grading, hepatic fat accumulation was evaluated using low- to medium-power magnification estimation of hepatocyte involvement by macrosteatosis or microsteatosis with grade 0 at <5% of parenchyma, grade 1 at 5%–33%, grade 2 at 33%–66%, and grade 3 at >66%.

Clinical variables were obtained from an electronic database as described previously [19]. Briefly, clinical data were extracted from enterprise-level data warehouse which contains a variety of data from the Geisinger electronic health record (EpicCare EHR; Verona, WI). Variables extracted and used for analysis included basic clinical measures, demographics, diagnostic ICD-9 codes, medical and medication history, and common lab results (Supplementary Table S1) (see Supplementary Material available online at http://dx.doi.org/10.1155/2014/368210). The research was approved by the Geisinger Clinic Institutional Review Board and all participants provided written informed consent.

### 2.2. Statistical Analysis

Mean with interquartile range and overall range was used to describe the distribution of quantitative variables. Logistic regression was used to determine which of the more than 200 preoperative clinical variables were independently associated with the presence of any degree of hepatic lipid versus normal histology. In separate analyses, logistic regression was used to determine which preoperative clinical variables were independently associated with grade 1 steatosis versus grade 0 steatosis, that is, mild versus normal, and which variables were associated with grade 2 and grade 3 steatosis versus grade 1 steatosis, that is, severe versus mild. For all analyses, univariate analysis was conducted first to identify a subset of variables for consideration in model building; that is, those variables with univariate *P* values <0.10 were considered for subsequent inclusion in the model.

Regression modeling was conducted in two phases. First, variables were selected based on (1) whether statistical significance was achieved in univariate analysis and (2) whether published information was available implicating them in NAFLD/NASH. For the second phase, the variables significant in univariate analysis but not included in the first phase were each added individually to determine their effect on the association strength. Potential nonlinearity of continuous variables, for example, lab results, was evaluated through various transformations including the use of quartiles and categorization based on clinically relevant thresholds, for example, classifying glucose values by potentially diagnostic groups, <100 mg/dL, 100–124 mg/dL, and ≥125 mg/dL. The homeostasis model assessment of insulin resistance (HOMA-IR) was calculated using the equation [20]:

(1) HOMA-IR=fasting  glucose  (mmol/L)×fasting  insulin  (μU/mL)22.5.

Effect modification was determined with interaction terms between variables within the final multiple regression models. c-statistics (area under the curve) and receiver operator characteristics (ROC) curves were used to evaluate discrimination of the final multiple regression models. SAS version 9.3 was used for statistical analysis and *P* values < 0.05 were considered significant.

## 3. Results

### 3.1. Cohort Characteristics

We analyzed clinical and pathology data on 3209 patients with BMI >35 kg/m^2^ who had no evidence of HBV, HCV, or HIV and no diagnosis of alcohol abuse and who had undergone primary bariatric surgery [19]. Pathology data from intraoperative liver biopsy were available for 2970 patients (92%). The primary reason for absence of pathology results was lack of clinical consent for liver biopsy. Those with liver pathology results were more likely to be female (81% versus 75% male, *P* = 0.035) and slightly younger (45.7 years versus 47.6 years, *P* = 0.012) but with similar levels of body mass index (BMI) of 49.5 kg/m^2^ versus 49.6 kg/m^2^ (*P* = 0.897) and liver function test (LFT) results (AST: 26.3 versus 26.7 U/L, *P* = 0.644; ALT: 30.7 versus 29.0 U/L, *P* = 0.219).

### 3.2. Hepatic Fat

**Figure 1:**
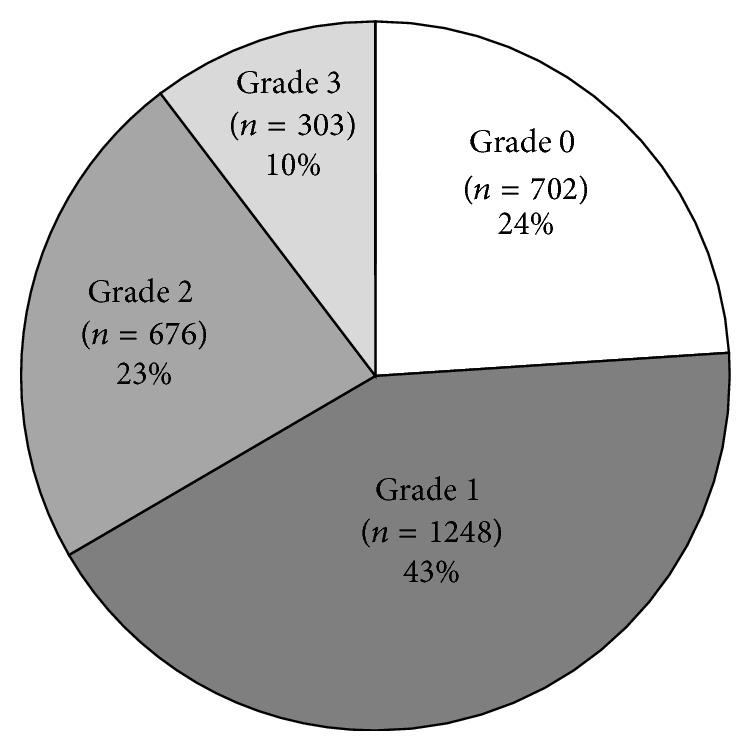
Distribution of hepatic fat by steatosis grades (*n* = 2929).

Of the 2970 patients with available liver pathology results, 41 (1%) were excluded from analysis due to adverse histological findings, that is, 7 with inflammation, 33 with fibrosis, and 1 with cirrhosis, in the setting of grade 0 hepatic fat. In the remaining 2929 patients (Table 1), the distribution of steatosis (Figure 1) was 24% grade 0, 43% grade 1, 23% grade 2, and 10% grade 3.

### 3.3. Association Analyses

**Figure 2:**
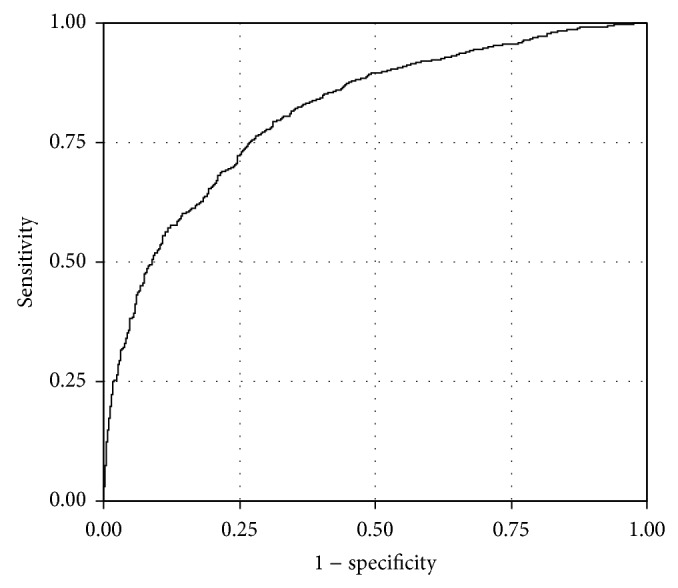
Receiver operator characteristic (ROC) curve for accuracy of final regression model for grade 0 versus grades 1, 2, and 3. The area under the curve was 0.8130.

In a univariate analysis comparing the 702 patients with grade 0 hepatic fat to the 2227 patients with mild, moderate, and severe hepatic fat accumulation, 87 of the 204 clinical variables were selected for the initial multiple regression model. These 87 variables were included in stepwise model building, resulting in 19 variables that demonstrated independent association (Table 2) and resulted in an overall area under the curve (c-statistic) of 0.813 (Figure 2). Ten laboratory measures were associated with presence of hepatic fat including glucose level > 200 mg/dL, insulin level > 17 *μ*U/mL, triglycerides ≥ 125 mg/dL, HDL < 50 mg/dL, and an increasing odds ratio for increasing ALT levels. In addition, ferritin levels of more than 400 ng/mL for males or more than 100 ng/mL for females as well as red blood cell count of more than 4.38 M/uL, chloride of more than 103 mmol/L, iron binding capacity of at least 320 mcg/dL, and a serum zinc level of more than 70 mcg/dL were associated with a greater risk for hepatic fat. Use of the medications metformin, benzodiazepine anticonvulsants, and topical corticosteroids was also associated with an increased risk. Clinical variables including a waist-height ratio of more than 0.9, a clinical diagnosis of sleep apnea, and failing to lose at least 10% of excess body weight in the preoperative period also placed patients at increased risk. Only two variables, the use of estrogen and/or progestin or serum creatinine level of >1.2 mg/dL, were associated with a decreased risk for hepatic fat. Interaction terms between these measures were not significant, suggesting that the effects are consistent within selected subsets of the population.

The above analysis was repeated comparing grade 1 (mild) to grade 0, which produced essentially identical results (data not shown). A reanalysis was also conducted for the patients who were documented to have no use of alcohol. In this subgroup, the overall area under the curve (c-statistic) increased slightly to 0.826, although the no alcohol group had a higher percentage of T2D that may have also influenced hepatic fat content. Similarly, the HOMA-IR was used instead of glucose and insulin, which slightly increased the area under the curve (c-statistic) to 0.817.

**Figure 3:**
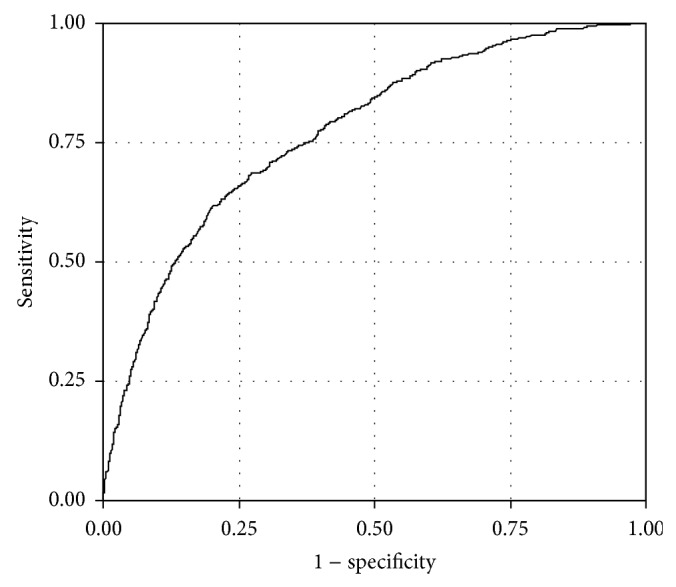
Receiver operator characteristic (ROC) curve for accuracy of final regression model for grades 2 and 3 steatosis versus grade 1 steatosis. The area under the curve was 0.7724.

We then sought to determine what clinical factors were associated with higher levels of hepatic fat versus low levels by comparing moderate (grade 2) and severe (grade 3) to mild (grade 1). For this analysis, the 702 patients with grade 0 hepatic fat levels were excluded from the analysis, leaving 2227. In univariate analysis, the 1248 patients with grade 1 were compared to the 979 patients with grades 2 and 3. A total of 56 of the 204 clinical variables were brought forward for multiple regression analysis. These 56 variables were included in stepwise model building, resulting in 11 variables that were independently associated with the higher level (grades 2 and 3) of hepatic fat (Table 3), including 7 that overlapped with those associated with the presence of any fat. The model resulted in an overall area under the ROC curve (c-statistic) of 0.772 (Figure 3). The 7 variables in common to the two regression models included serum glucose, though significant at a lower threshold (greater than 100 mg/dL) with severity of hepatic fat and increasing to more than 2.5-fold risk at levels 200 mg/dL or higher. Similarly an increased risk was found for insulin (>17 *μ*U/mL), triglycerides (≥125 mg/dL), and use of metformin. Lower preoperative weight loss was again associated with increased risk. A graded increased risk was associated with ALT levels to more than 5-fold at ALT >37 U/L, while AST levels >31 U/L showed an almost 1.7-fold increased risk. Independent factors increasing the odds of moderate to severe hepatic fat included female gender (30% increase), patients with a BUN level <13 mg/dL (>50% increase), and use of tricyclic or modified cyclic antidepressants (50% increase) or fibric acid derivatives (>75% increase). Interaction terms between these measures were not significant (data not shown). We also evaluated the association in patients who were documented to have no use of alcohol. In this subgroup, the overall area under the curve (c-statistic) increased slightly to 0.785. Reanalysis using HOMA-IR increased the area under the curve (c-statistic) to 0.777.

## 4. Discussion

Fatty liver is a highly prevalent finding in extreme obesity that has several potentially significant clinical sequelae. The characterization of associated clinical characteristics in populations with high prevalence, such as those with extreme obesity, is thus important for identifying potentially modifiable risk factors and putative pathophysiological mechanisms. To date, most studies have been limited by small sample sizes, use of noninvasive methods to assess hepatic fat, and a focus on a relatively small number of clinical variables. We surveyed 22 published reports (Supplementary References) on hepatic fat accumulation representing 4274 patients with extreme obesity undergoing bariatric surgery and liver biopsy (Supplementary Table S2), a prime opportunity in which to assess population-based aspects since such patients generally are not documented to have, nor are suspected of having, liver disease, which is a bias for studies based on percutaneous liver biopsies. Our single center study thus represents a sample size of nearly 75% of that of the combined reports we were able to identify. Our basic cohort characteristics were similar to the 78% female, average age of 41.5 years, average BMI of 47.6 kg/m^2^, and 75% steatosis prevalence reported in these studies (Supplementary Table S2). The overall prevalence of hepatic fat in these studies is slightly lower than previously reported [5], likely due to the use of stricter histological criteria [18] in the more recent studies we included.

The accuracy of our final multiple logistic regression model for presence of hepatic fat was over 80%. The variables contributing to this moderate level of accuracy included elevated AST and/or ALT, where risk progressively increased over a relatively restricted range (by quartile) considered “normal,” from <20 U/L to greater than 37 U/L. These data are consistent with that reported in other studies [21] and a progressive but low level of hepatocyte injury due to triglyceride accumulation. Older age also had a slight effect on risk, consistent with previous studies [9]. The association of fasting plasma glucose, insulin, and biguanide use with the presence of hepatic lipid is concordant with previous studies identifying insulin resistance/type 2 diabetes as a risk factor [7]. However, whether hepatic fat is a consequence or cause of insulin resistance/type 2 diabetes is controversial. Accumulating data support a role for hepatic diacylglycerols in the pathogenesis of hepatic insulin resistance [3, 22].

Fatty liver has been independently associated with a lower level of HDL cholesterol and a higher serum triglyceride level, similar to our findings, although large studies have used imaging as a surrogate for classifying hepatic fat [23, 24]. Interestingly, no associations with LDL cholesterol were found, which we also report here. However, a substantial proportion (36%) of the patients we analyzed were prescribed lipid-lowering agents. Although such drugs, that is, statins, have generally been found to cause an amelioration of liver fat, noninvasive methods were used in most studies, which also lacked rigorous study designs [25]. Statins are not recommended as a treatment option specifically for fatty liver in recent clinical guidelines [26].

We found that patients with a diagnosis of obstructive sleep apnea had more severe levels of hepatic fat accumulation, replicating findings from recent meta-analyses [27]. This intriguing association does not appear to be due to simple hypoxic insult to the liver [28]. The pathophysiology is unclear and is based primarily on animal studies, although lipogenic genes appear to be upregulated from intermittent hypoxia, which may be mediated by activation of hypoxia inducible factors in the liver through an increase in circulating free fatty acids released from hypoxic adipocytes. Further studies will be needed to further define the potential role of sleep apnea as a risk factor.

Higher ferritin, iron binding capacity, red blood cell count, and serum zinc levels were also found to be associated with hepatic fat accumulation. The ferritin, iron binding capacity, and red cell count are consistent with a dysregulation of iron metabolism that has previously been described [29], given that the liver is a primary storage site for iron. In an animal model, increased hepatic iron content preceded fat accumulation and insulin resistance in response to a high fat diet [30], suggesting that an increase in iron may play a mechanistic role in the role of hepatic fat in modulating glucose metabolism. Zinc levels have been related to fatty liver secondary to alcohol consumption [31] and in tetracycline-induced [32] fatty liver in animal models but not with diet-induced fatty liver.

Female sex and the use of estrogens and progestins (a surrogate for female sex) were associated with slightly increased odds for hepatic fat, consistent with previous human and animal studies [33]. Several drug classes including tricyclic antidepressants and fibric acid derivatives also increased the odds, a known effect of certain drugs [34]. Animal model data support a role for certain older tricyclic or modified cyclic antidepressants in fatty liver [35] and, contrary to our findings, a decreased risk for fibric acid derivatives [36]. At present, data from human studies are limited.

Despite the large sample size, our data were limited to a single center with patients who were primarily of Caucasian European ancestry and were predominantly middle-aged women, limiting generalizability of the data, though allowing for comparison to other bariatric surgery studies. The extreme level of obesity limits extension of the findings to lower BMI levels. A cohort of bariatric surgery patients may also be a biased population relative to the vast majority of individuals with extreme obesity who do not undergo the procedure.

We characterized histologically defined fatty liver in the context of the largest prospectively enrolled single center cohort of extremely obese adults coupled to a comprehensive and well-curated entry database of clinical data [19]. Regression modeling identified known and novel factors associated with hepatic fat accumulation that provide support for further studies on their potential role in fatty liver in extreme obesity.

## Supplementary Material

The Supplemental materials contain Table S1 that contains the clinical variables used in regression analysis (n=204 variables) and Table S2 that contains a summary of 22 available published reports on 4274 patients with extreme obesity undergoing bariatric surgery and liver biopsy.

## Figures and Tables

**Table 1:** The demographics of the 2929 patients who were included in the study population.

Age, years	Median (IQR^*^)	46 (37–45)
	Range	[18, 75]

Gender	Female, % (*n*)	81% (*n* = 2360)
	Male, % (*n*)	19% (*n* = 569)

Race	White, % (*n*)	97% (*n* = 2848)
	Black, % (*n*)	2% (*n* = 64)
	Other/unknown, % (*n*)	1% (*n* = 17)

BMI, kg/m^2^	Median (IQR^*^)	47.7 (43.1–54.1)
	[Range]	[35.0, 98.4]

Alcohol use	Yes, % (*n*)	38% (*n* = 998)
	No, % (*n*)	62% (*n* = 1646)
	Unknown	*N* = 285

Smoking history	Current/quit, % (*n*)	35% (*n* = 782)
	Never, % (*n*)	65% (*n* = 1430)
	Unknown	*N* = 717

Diabetes	Yes, % (*n*)	35% (*n* = 1038)

Hypertension	Yes, % (*n*)	50% (*n* = 1461)

Dyslipidemia	Yes, % (*n*)	40% (*n* = 1162)

^*^Interquartile range.

**Table 2:** Multiple regression results for presence of hepatic fat (grades 0, 1, 2, and 3 versus grade 0).

	Odds ratio	95% confidence interval	*P* value
Glucose level			
<100 mg/dL	Reference		
100–124 mg/dL	1.008	[0.774, 1.313]	0.950
125–199 mg/dL	1.447	[0.976, 2.145]	0.656
200+ mg/dL	3.153	[1.482, 6.709]	0.0029
Serum insulin > 17 *µ*U/mL	2.097	[1.692, 2.600]	<0.0001
Triglyceride level			
<125 mg/dL	Reference		
125–199 mg/dL	1.298	[1.037, 1.625]	0.0227
200+ mg/dL	2.090	[1.535, 2.845]	<0.0001
HDL < 50 mg/dL	1.479	[1.198, 1.828]	0.0003
ALT level			
Quartile 1 (<20 U/L)	Reference		
Quartile 2: (20–25 U/L)	1.470	[1.142, 1.891]	0.0027
Quartile 3: (26–36 U/L)	2.286	[1.747, 2.992]	<0.0001
Quartile 4: (37+ U/L)	3.233	[2.364, 4.422]	<0.0001
Elevated ferritin (>400 ng/mL M, >100 ng/mL F)	1.654	[1.210, 2.261]	0.0016
Creatinine level			
Low (<0.6 mg/dL)	1.571	[0.926, 2.663]	0.0939
Normal (0.6–1.1 mg/dL)	Reference		
High (1.2+ mg/dL)	0.389	[0.261, 0.581]	<0.0001
Red blood cell count			
Quartile 1 (<4.38 M/uL)	Reference		
Quartile 2, 3, 4 (4.38+ M/uL)	1.350	[1.078, 1.690]	0.0088
Chloride level			
Quartile 1 or 2 (<103 mmol/L)	1.454	[1.185, 1.784]	0.0003
Quartile 3 or 4 (103+ mmol/L)	Reference		
Iron bind. cap. level			
Quartile 1 or 2 (<320 mcg/dL)	Reference		
Quartile 3 or 4 (320+ mcg/dL)	1.332	[1.071, 1.657]	0.0099
Zinc level			
Quartile 1 (<70 mcg/dL)	Reference		
Quartile 2, 3, or 4 (70+ mcg/dL)	1.351	[1.024, 1.781]	0.0332
Use of metformin	2.041	[1.634, 2.551]	<0.0001
Use of estrogen/progestin	0.427	[0.308, 0.592]	<0.0001
Use of benzodiazepine anticonvulsants	1.855	[1.050, 3.278]	0.0333
Use of topical corticosteroids	1.521	[1.057, 2.189]	0.0238
Age > 40	1.295	[1.030, 1.629]	0.0272
Waist height ratio			
<0.70	Reference		
0.70–0.90	1.450	[1.059, 1.985]	0.0204
>0.90	2.201	[1.452, 3.336]	0.0002
Sleep apnea diagnosis	1.481	[1.198, 1.829]	0.0003
Low pre-op weight loss (<10% EWL)	1.844	[1.507, 2.256]	<0.0001

c-statistic = 0.813.

**Table 3:** Multiple regression results for severity of hepatic fat (grades 2 and 3 versus grade 1).

	Odds ratio	95% confidence interval	*P* value
Glucose level			
<100 mg/dL	Reference		
100–124 mg/dL	1.475	[1.161, 1.876]	0.0015
125–199 mg/dL	1.655	[1.231, 2.225]	0.0009
200+ mg/dL	2.530	[1.632, 3.922]	<0.0001
Serum insulin > 17 *µ*U/mL	1.900	[1.532, 2.357]	<0.0001
Triglyceride level			
<125 mg/dL	Reference		
125–199 mg/dL	1.415	[1.126, 1.778]	0.0029
200+ mg/dL	1.448	[1.117, 1.877]	0.0052
ALT level			
Quartile 1 (<20 U/L)	Reference		
Quartile 2: (20–25 U/L)	1.690	[1.247, 2.290]	0.0007
Quartile 3: (26–36 U/L)	2.765	[2.065, 3.703]	<0.0001
Quartile 4: (37+ U/L)	5.144	[3.616, 7.319]	<0.0001
AST level			
Quartile 1, 2, or 3 (<31 U/L)	Reference		
Quartile 4 (31+ U/L)	1.696	[1.288, 2.235]	0.0002
BUN level			
Quartile 1 (<13 mg/dL)	1.556	[1.264, 1.917]	<0.0001
Quartile 2, 3, or 4 (13+ mg/dL)	Reference		
Use of metformin	1.432	[1.175, 1.745]	0.0004
Use of fibric acid derivative	1.766	[1.188, 2.626]	0.0050
Use of tricyclics or modified cyclics	1.488	[1.099, 2.013]	0.0101
Female gender	1.295	[1.017, 1.649]	0.0359
Pre-op weight change			
Weight gain	3.325	[2.288, 4.834]	<0.0001
0–4 %EWL	2.636	[1.793, 3.876]	<0.0001
5–9 %EWL	2.436	[1.703, 3.484]	<0.0001
10–19 %EWL	1.561	[1.115, 2.185]	0.0094
20+ %EWL	Reference		

c-statistic = 0.772.

## References

[1] Wang Y., Li Y. Y., Nie Y. Q., Zhou Y. J., Cao C. Y., Xu L. (2013). Association between metabolic syndrome and the development of non-alcoholic fatty liver disease. *Experimental and Therapeutic Medicine*.

[2] Fabbrini E., Sullivan S., Klein S. (2010). Obesity and nonalcoholic fatty liver disease: biochemical, metabolic, and clinical implications. *Hepatology*.

[3] Birkenfeld A. L., Shulman G. I. (2014). Nonalcoholic fatty liver disease, hepatic insulin resistance, and type 2 diabetes. *Hepatology*.

[4] Day C. P. (2006). Genes or environment to determine alcoholic liver disease and non-alcoholic fatty liver disease. *Liver International*.

[5] Machado M., Marques-Vidal P., Cortez-Pinto H. (2006). Hepatic histology in obese patients undergoing bariatric surgery. *Journal of Hepatology*.

[6] Naik A., Belic A., Zanger U. M., Rozman D. (2013). Molecular interactions between NAFLD and xenobiotic metabolism. *Frontiers in Genetics*.

[7] Bugianesi E., Moscatiello S., Ciaravella M. F., Marchesini G. (2010). Insulin resistance in nonalcoholic fatty liver disease. *Current Pharmaceutical Design*.

[8] Vernon G., Baranova A., Younossi Z. M. (2011). Systematic review: the epidemiology and natural history of non-alcoholic fatty liver disease and non-alcoholic steatohepatitis in adults. *Alimentary Pharmacology & Therapeutics*.

[9] Bhala N., Angulo P., van der Poorten D., Lee E., Hui J. M., Saracco G. (2011). The natural history of nonalcoholic fatty liver disease with advanced fibrosis or cirrhosis: an international collaborative study. *Hepatology*.

[10] Charlton M. (2013). Evolving aspects of liver transplantation for nonalcoholic steatohepatitis. *Current Opinion in Organ Transplantation*.

[11] Hashimoto E., Taniai M., Tokushige K. (2013). Characteristics and diagnosis of NAFLD/NASH. *Journal of Gastroenterology and Hepatology*.

[12] Gabrielli M., Moisan F., Vidal M. (2012). Steatotic livers. Can we use them in OLTX? Outcome data from a prospective baseline liver biopsy study. *Annals of Hepatology*.

[13] Sharma A., Ashworth A., Behnke M., Cotterell A., Posner M., Fisher R. A. (2013). Donor selection for adult-to-adult living donor liver transplantation: well begun is half done. *Transplantation*.

[14] Miyake T., Kumagi T., Furukawa S. (2013). Non-alcoholic fatty liver disease: factors associated with its presence and onset. *Journal of Gastroenterology and Hepatology*.

[15] Still C. D., Craig Wood G., Chu X., Manney C. (2013). Clinical factors associated with weight loss outcomes after Roux-en-Y gastric bypass surgery. *Obesity*.

[16] Association A. P. (2000). *Diagnostic and Statistical Manual of Mental Disorders*.

[17] Gorden A., Yang R., Yerges-Armstrong L. M. (2013). Genetic variation at NCAN locus is associated with inflammation and fibrosis in non-alcoholic fatty liver disease in morbid obesity. *Human Heredity*.

[18] Kleiner D. E., Brunt E. M., Van Natta M. (2005). Design and validation of a histological scoring system for nonalcoholic fatty liver disease. *Hepatology*.

[19] Wood G. C., Chu X., Manney C. (2012). An electronic health record-enabled obesity database. *BMC Medical Informatics and Decision Making*.

[20] Matthews D. R., Hosker J. P., Rudenski A. S., Naylor B. A., Treacher D. F., Turner R. C. (1985). Homeostasis model assessment: insulin resistance and beta-cell function from fasting plasma glucose and insulin concentrations in man. *Diabetologia*.

[21] Kim K. S., Oh H. J., Kim D. J. (2013). The association between non-alcoholic fatty liver disease and carotid atherosclerosis in subjects with within-reference range alanine aminotransferase levels. *Endocrine Journal*.

[22] Kumashiro N., Erion D. M., Zhang D. (2011). Cellular mechanism of insulin resistance in nonalcoholic fatty liver disease. *Proceedings of the National Academy of Sciences of the United States of America*.

[23] DeFilippis A. P., Blaha M. J., Martin S. S. (2013). Nonalcoholic fatty liver disease and serum lipoproteins: the multi-ethnic study of atherosclerosis. *Atherosclerosis*.

[24] Speliotes E. K., Massaro J. M., Hoffmann U., Vasan R. S., Meigs J. B., Sahani D. V. (2010). Fatty liver is associated with dyslipidemia and dysglycemia independent of visceral fat: the Framingham Heart Study. *Hepatology*.

[25] Chatrath H., Vuppalanchi R., Chalasani N. (2012). Dyslipidemia in patients with nonalcoholic fatty liver disease. *Seminars in Liver Disease*.

[26] Chalasani N., Younossi Z., Lavine J. E. (2012). The diagnosis and management of non-alcoholic fatty liver disease: practice Guideline by the American Association for the Study of Liver Diseases. *Hepatology*.

[27] Sookoian S., Pirola C. J. (2013). Obstructive sleep apnea is associated with fatty liver and abnormal liver enzymes: a meta-analysis. *Obesity Surgery*.

[28] Mirrakhimov A. E., Polotsky V. Y. (2012). Obstructive sleep apnea and non-alcoholic fatty liver disease: is the liver another target?. *Frontiers in Neurology*.

[29] Aigner E., Theurl I., Theurl M. (2008). Pathways underlying iron accumulation in human nonalcoholic fatty liver disease. *The American Journal of Clinical Nutrition*.

[30] Tsuchiya H., Ebata Y., Sakabe T., Hama S., Kogure K., Shiota G. (2013). High-fat, high-fructose diet induces hepatic iron overload via a hepcidin-independent mechanism prior to the onset of liver steatosis and insulin resistance in mice. *Metabolism*.

[31] Kang X., Zhong W., Liu J. (2009). Zinc supplementation reverses alcohol-induced steatosis in mice through reactivating hepatocyte nuclear factor-4alpha and peroxisome proliferator-activated receptor-alpha. *Hepatology*.

[32] Mikhail T. H., Nicola W. G., Ibrahim K. H., Salama S. H., Emam M. (1996). Abnormal zinc and copper metabolism in hepatic steatosis. *Bollettino Chimico Farmaceutico*.

[33] Lavoie J.-M., Pighon A. (2012). NAFLD, estrogens, and physical exercise: the animal model. *Journal of Nutrition and Metabolism*.

[34] Amacher D. E. (2011). The mechanistic basis for the induction of hepatic steatosis by xenobiotics. *Expert Opinion on Drug Metabolism & Toxicology*.

[35] Letteron P., Sutton A., Mansouri A., Fromenty B., Pessayre D. (2003). Inhibition of microsomal triglyceride transfer protein: another mechanism for drug-induced steatosis in mice. *Hepatology*.

[36] Karahashi M., Hoshina M., Yamazaki T. (2013). Fibrates reduce triacylglycerol content by upregulating adipose triglyceride lipase in the liver of rats. *Journal of Pharmacological Sciences*.

