# Supplementary material for: Identification of Novel Clinical Factors Associated with Hepatic Fat Accumulation in Extreme Obesity
Source: J Obes. 2014 Dec 24;2014:368210. doi: 10.1155/2014/368210 (PMC4290025; doi:10.1155/2014/368210)
Supplement: Supplementary file 1 — The Supplemental materials contain Table S1 that contains the clinical variables used in regression analysis (n=204 variables) and Table S2 that contains a summary of 22 available published reports on 4274 patients with extreme obesity undergoing bariatric surgery and liver biopsy. [file 368210.f1.pdf]

Supplementary Information

Identification of novel clinical factors associated with hepatic fat accumulation in extreme obesity.

Gerhard, et al.

The Supplemental materials have the following sections in order:

1. Table S1. Clinical variables used in regression analysis (n=204 variables).

2. Table S2. Summary of 22 available published reports on 4274 patients with extreme obesity undergoing bariatric surgery and liver biopsy.

3. References for Table S2.

Table 1: Clinical variables used in regression analysis (n=204 variables).

Baseline information and demographics

- Gender
- Ethnic group
- Age
- Height
- Weight at baseline
- BMI at baseline
- Waist circumference
- Waist-height ratio
- REE
- VO2max
- Alcohol use
- Smoking history
- Weight loss during pre-op program
- Time in pre-op program
- Surgical approach (open versus lap)
- Number of comorbidities during pre-op period
- Number of medications during pre-op period

Comorbidities on problem list from baseline to surgery using 3-digit ICD9, requiring 2% of population

- ICD_401
- ICD_272
- ICD_250
- ICD_780
- ICD_530
- ICD_311
- ICD_715
- ICD_244
- ICD_493
- ICD_724
- ICD_722
- ICD_300
- ICD_477
- ICD_346
- ICD_251
- ICD_305
- ICD_327
- ICD_296
- ICD_786
- ICD_729
- ICD_782
- ICD_309
- ICD_790
- ICD_719
- ICD_564
- ICD_256
- ICD_414
- ICD_553
- ICD_354
- ICD_789
- ICD_726
- ICD_784
- ICD_269
- ICD_592
- ICD_427
- ICD_626
- ICD_787
- ICD_268
- ICD_627
- ICD_429
- ICD_625
- ICD_276
- ICD_794
- ICD_274
- ICD_728
- ICD_357
- ICD_785
- ICD_307
- ICD_796
- ICD_428

Medications on reconciliation list from baseline to 1 month prior to surgery using subclass, requiring 2% of population

- Biguanides
- NSAID
- PPI
- SSRI
- Statins
- OpioidAgonist
- OpioidCombo
- OilSolVit
- ACE
- Salicylates
- Sympath
- BBCS
- H2
- LoopDiur
- ThyroidHorm
- AnalgOther
- MultiVit
- Insulin
- AntihistNonsed
- Calcium
- Thiazides
- Benzod
- MuscleRelax
- AntiHTNcomb
- NasalSteroids
- AnticonvMisc
- SNRI
- Sulfonylureas
- InsSensAgents
- CCB
- Potassium
- Glucocort
- ARB
- Cough_Cold_AllComb
- AnorexNonAmph
- AntidepressMisc
- MultiVitMinerals
- CorticTopical
- WaterSolubleVitamins
- Heparin
- Azithromycin
- Aminopenicillins
- NonBarbitHypnotics
- SteroidInhalants
- LeukotrieneModulators
- Tricyclic
- AntifungalsTopical
- Iron
- SmokingDeterrents
- ComboContraceptOral
- FibricAcid
- Fluoroquinolones
- Nitrates
- SerotoninAgonists
- AntiInfectiveMiscCombo
- Cephalosporins1stGen
- UrinaryAntispasmodics
- ModifiedCyclics
- PenicillinCombo
- DiureticCombo
- Tetracyclines
- AntiemetAnticholinergic
- AnticonvulsantsBenzod
- PlateAggrInhibitors
- Coumarin
- AntihyperlipidCombo
- PotSparingDiuretics
- AntiObesityAgents
- SurfactantLaxatives
- AntianxietyAgentsMisc
- ImidazoleRelAntifungal
- GoutAgents
- AntiparkinDopaminergic
- Cobalamins
- Dibenzapines
- Progestins
- IntesCholAbsorptionInhib
- Antitussives
- AntihistPhenothiazines
- Clarithromycin
- UlcerTherapyCombo
- Estrogens
- ProgestContratInjectable
- FolicAcidFolates
- BronchoAnticholinergics
- BulkLaxatives
- AntiInfectiveAgentsMisc
- AntiadrenergicAntiHTN
- BBNS
- GastrointestlStimulants
- AnalgesicCombo
- IncretinMimetAgGLP1

Labs prior to surgery found in >50% of population

- glucose
- insulin
- hba1c
- bun
- sodium
- potassium
- CHLORIDE
- CO2
- creat
- calcium
- GFR
- WBC
- RBC
- HEMOGLOBIN
- HCT
- MCV
- MCH
- MCHC
- RDW
- PLATELET
- TSH
- TRIG
- CHOL
- HDL
- LDL
- CholHDLRat
- ALT
- AST
- AlkPhos
- TotBili
- MPV
- AnionGap
- Ferritin
- Iron
- IBC
- TransSat
- PTH
- FolicAcid
- Vitamin D 25 OH D2
- Vitamin D 25 OH D3
- Vitamin D 25 TOTAL
- Creatinine, RD Urine
- Protein, RD Urine
- Prot/Creat Ratio
- Zinc level

Reference	Year	Sample size	% Female	Age (years +/- SD)	BMI (KG/M2 +/- SD)	Normal (%)	Steatosis (%)
(1)	1998	528	81	36 ± 11	43 ± 7	21%	74%
(2)	1999	551	80	36 ± 9	47 ± 9	14%	86%
(3)	2004	105	78	41 ± 11	47 ± 7	4%	71%
(4)	2002	35	74	33 ± 10	44 ± 8	9%	-
(5)	2003	48	69	42 ± 10	59.9 ± 12	15%	85%
(6)	2003	48	84	-	51 ± 2	12%	58%
(7)	2004	195	88	41 ± 9	48 ± 7	33%	67%
(8)	2005	212	80	42 ± 10	48 ± 9	7%	93%
(9)	2005	125	62	40 ± 11	42 ± 6	37%	63%
(10)	2005	112	74	36 ± 10	49 ± 8	1%	99%
(11)	2005	51	-	-	53 ± 1	2%	98%
(12)	2007	135	80	46 ± 10	50 ± 8	14%	59%
(13)	2007	137	-	-	-	4%	92%
(14)	2009	142	79	49 ± 10	48 ± 7	30%	70%
(15)	2009	238	75	42 ± 9	56 ± 10	11%	89%
(16)	2010	129	81	-	-	-	26%

(17)	2010	679	80	43	48 ± 8	16%	77%
(18)	2011	437	78	48 ± 11	49 ± 9	22%	78%
(19)	2012	21	62	30 ± 8	-	10%	90%
(20)	2012	26	-	-	49 ± 5	-	-
(21)	2013	24	88	38 ± 7	51 ± 11	-	33%
(22)	2014	296	61	47 ± 9	44 ± 9	-	42%

1. Luyckx FH, Desaive C, Thiry A, Dewe W, Scheen AJ, Gielen JE, Lefebvre PJ. Liver abnormalities in severely obese subjects: effect of drastic weight loss after gastroplasty. Int J Obes Relat Metab Disord 1998;22:222-226.
2. Marceau P, Biron S, Hould FS, Marceau S, Simard S, Thung SN, Kral JG. Liver pathology and the metabolic syndrome X in severe obesity. J Clin Endocrinol Metab 1999;84:1513-1517.
3. Dixon JB, Bhathal PS, Hughes NR, O'Brien PE. Nonalcoholic fatty liver disease: Improvement in liver histological analysis with weight loss. Hepatology 2004;39:1647-1654.
4. Sepulveda-Flores RN, Vera-Cabrera L, Flores-Gutierrez JP, Maldonado-Garza H, Salinas-Garza R, Zorrilla-Blanco P, Bosques-Padilla FJ. Obesity-related non-alcoholic steatohepatitis and TGF-beta1 serum levels in relation to morbid obesity. Ann Hepatol 2002;1:36-39.
5. Beymer C, Kowdley KV, Larson A, Edmonson P, Dellinger EP, Flum DR. Prevalence and predictors of asymptomatic liver disease in patients undergoing gastric bypass surgery. Arch Surg 2003;138:1240-1244.
6. Spaulding L, Trainer T, Janiec D. Prevalence of non-alcoholic steatohepatitis in morbidly obese subjects undergoing gastric bypass. Obes Surg 2003;13:347-349.
7. Abrams GA, Kunde SS, Lazenby AJ, Clements RH. Portal fibrosis and hepatic steatosis in morbidly obese subjects: A spectrum of nonalcoholic fatty liver disease. Hepatology 2004;40:475-483.
8. Ong JP, Younossi ZM. Approach to the diagnosis and treatment of nonalcoholic fatty liver disease. Clin Liver Dis 2005;9:617-634, vi.
9. Boza C, Riquelme A, Ibanez L, Duarte I, Norero E, Viviani P, Soza A, et al. Predictors of nonalcoholic steatohepatitis (NASH) in obese patients undergoing gastric bypass. Obes Surg 2005;15:1148-1153.
10. Lima ML, Mourao SC, Diniz MT, Leite VH. Hepatic histopathology of patients with morbid obesity submitted to gastric bypass. Obes Surg 2005;15:661-669.
11. Stratopoulos C, Papakonstantinou A, Terzis I, Spiliadi C, Dimitriades G, Komesidou V, Kitsanta P, et al. Changes in liver histology accompanying massive weight loss after gastroplasty for morbid obesity. Obes Surg 2005;15:1154-1160.
12. Kroh M, Liu R, Chand B. Laparoscopic bariatric surgery: what else are we uncovering? Liver pathology and preoperative indicators of advanced liver disease in morbidly obese patients. Surg Endosc 2007;21:1957-1960.
13. Jouet P, Sabate JM, Maillard D, Msika S, Mechler C, Ledoux S, Harnois F, et al. Relationship between obstructive sleep apnea and liver abnormalities in morbidly obese patients: a prospective study. Obes Surg 2007;17:478-485.
14. Kashyap SR, Diab DL, Baker AR, Yerian L, Bajaj H, Gray-McGuire C, Schauer PR, et al. Triglyceride levels and not adipokine concentrations are closely related to severity of nonalcoholic fatty liver disease in an obesity surgery cohort. Obesity (Silver Spring) 2009;17:1696-1701.
15. Kallwitz ER, Guzman G, TenCate V, Vitello J, Layden-Almer J, Berkes J, Patel R, et al. The histologic spectrum of liver disease in African-American, non-Hispanic white, and Hispanic obesity surgery patients. Am J Gastroenterol 2009;104:64-69.

16. Guajardo-Salinas GE, Hilmy A. Prevalence of nonalcoholic fatty liver disease (NAFLD) and utility of FIBROspect II to detect liver fibrosis in morbidly obese Hispano-American patients undergoing gastric bypass. Obes Surg 2010;20:1647-1653.
17. Bedossa P, Poitou C, Veyrie N, Bouillot JL, Basdevant A, Paradis V, Tordjman J, et al. Histopathological algorithm and scoring system for evaluation of liver lesions in morbidly obese patients. Hepatology 2012;56:1751-1759.
18. Ribeireiro T, Swain J, Sarr M, Kendrick M, Que F, Sanderson S, Krishnan A, et al. NAFLD and insulin resistance do not increase the risk of postoperative complications among patients undergoing bariatric surgery--a prospective analysis. Obes Surg 2011;21:310-315.
19. Tai CM, Huang CK, Hwang JC, Chiang H, Chang CY, Lee CT, Yu ML, et al. Improvement of nonalcoholic fatty liver disease after bariatric surgery in morbidly obese Chinese patients. Obes Surg 2012;22:1016-1021.
20. Vargas V, Allende H, Lecube A, Salcedo MT, Baena-Fustegueras JA, Fort JM, Rivero J, et al. Surgically induced weight loss by gastric bypass improves non alcoholic fatty liver disease in morbid obese patients. World J Hepatol 2012;4:382-388.
21. Holterman AX, Guzman G, Fantuzzi G, Wang H, Aigner K, Browne A, Holterman M. Nonalcoholic fatty liver disease in severely obese adolescent and adult patients. Obesity (Silver Spring) 2013;21:591-597.
22. Hyysalo J, Mannisto VT, Zhou Y, Arola J, Karja V, Leivonen M, Juuti A, et al. A population-based study on the prevalence of NASH using scores validated against liver histology. J Hepatol 2013.
